# Iatrogenic Pneumopericardium After Pericardiocentesis: A Systematic Review and Case Report

**DOI:** 10.3390/jcdd12070246

**Published:** 2025-06-26

**Authors:** Andreas Merz, Hong Ran, Cheng-Ying Chiu, Henryk Dreger, Daniel Armando Morris, Matthias Schneider-Reigbert

**Affiliations:** 1 Deutsches Herzzentrum der Charité, Department of Cardiology, Angiology and Intensive Care Medicine, 13353 Berlin, Germany; 2 DZHK (German Center for Cardiovascular Research), Partner Site Berlin, 10785 Berlin, Germany; 3 Charité—Universitätsmedizin Berlin, Corporate Member of Freie Universität Berlin and Humboldt-Universität zu Berlin, 10117 Berlin, Germany; 4 Department of Echocardiography, Nanjing First Hospital, Nanjing Medical University, Nanjing 210006, China

**Keywords:** pneumopericardium, pericardiocentesis, echocardiography, air gap, swirling bubble, case report, systematic review

## Abstract

Background: Pneumopericardium is the presence of air within the pericardial cavity. We report a case of iatrogenic pneumopericardium following pericardiocentesis in a patient with primary cardiac angiosarcoma. Additionally, we provide a systematic review of pericardiocentesis-associated pneumopericardium to offer a comprehensive overview and evaluate the role of echocardiography in its diagnosis. Methods: The PubMed database was searched from inception until January 2025 to perform a systematic review following the Preferred Reporting Items for Systematic Reviews and Meta-Analyses (PRISMA) guidelines to evaluate articles on iatrogenic pneumopericardium following pericardiocentesis published in the English language. The Joanna Briggs Institute (JBI) Critical Appraisal Checklist for Case Reports was used to appraise the included case reports. Results: Of the 108 search results obtained, after screening and a backward citation search, 37 articles were selected for inclusion in this review, accounting for a total of 37 patients. According to the JBI Critical Appraisal Checklist for Case Reports, 7 case reports were of high quality and 12 of low quality. The overall evidence of quality of the case reports was moderate, and 51.6% of patients developed hemodynamic compromise or showed signs of cardiac tamponade. The main underlying cause for the development of pneumopericardium was issues relating to the catheter drainage system; 64.9% of cases required decompressive therapy. Conclusions: Pneumopericardium can occur as a complication after pericardiocentesis and must therefore be considered in symptomatic patients. While detection by transthoracic echocardiography is difficult and relies on non-validated signs, chest X-ray and computed tomography can provide a definitive diagnosis.

## 1. Introduction

Pneumopericardium is defined as the presence of air within the pericardial cavity. Its etiology is heterogeneous and is commonly related to trauma. Other causes include the presence of pericardial sac fistulas, pericarditis with gas-forming organisms, and patients on mechanical ventilation, as well as respiratory distress syndrome in neonates [1,2,3,4]. It may also occur iatrogenically. The intrapericardial instillation of air resulting in intentional pneumopericardium has been used to improve visibility during pericardioscopy [5] and for phrenic nerve protection during epicardial catheter ablation of arrhythmias [6], as well as for the treatment of tuberculous pericarditis with effusion [7]. Pneumopericardium rarely occurs accidentally after pericardiocentesis [8,9], where it can result from a direct pleuro-pericardial connection or reverse air leakage within or around the pericardial drainage system. Diagnosis is based on imaging, primarily relying on chest X-ray and computed tomography (CT), while detection through transthoracic echocardiography (TTE) can be challenging. The clinical presentation of pneumopericardium varies; signs and symptoms resemble the classical pericardial syndromes of pericardial effusion and, despite being uncommon, cardiac tamponade [10]. The management of pneumopericardium depends on both its etiology and clinical severity, the latter being determined by the quantity and rate of development [8,10]. It may resolve spontaneously or require prompt intervention if tension pneumopericardium occurs.

We report a rare case of iatrogenic pneumopericardium after pericardiocentesis in a patient with primary cardiac epithelioid angiosarcoma. Additionally, we provide a systematic review of the literature to offer a comprehensive overview of the clinical presentation, underlying causes, diagnosis, and management of patients with pneumopericardium following pericardiocentesis. Furthermore, we evaluate the role of TTE in the diagnosis of pericardiocentesis-associated pneumopericardium.

## 2. Case Report

A 51-year-old female patient was referred to our hospital for further investigation of a suspected cardiac tumor. One week prior, cardiac magnetic resonance imaging (CMR) had shown a 41 × 33 × 51 mm structure at the border of the right atrium (RA) and right ventricle (RV), with slight constriction of the tricuspid annulus, infiltration of the pericardium, and a moderate pericardial effusion. Five months prior to admission, she had undergone pericardiocentesis for pericardial effusion due to pericarditis. A follow-up CMR conducted one month later showed subacute perimyocarditis without signs of a cardiac tumor. Her medical history included pulmonary sarcoidosis and subclinical hypothyroidism. She was not on any regular medication. Her family history included a sister suffering from breast cancer.

Upon presentation, the patient reported symptoms of dyspnea (New York Heart Association functional class III) as well as occasional left-sided chest pain, aggravated by bending forward. Physical examination, vital signs, and laboratory results (including lactate dehydrogenase) were unremarkable. A thoracoscopic pericardial window was performed, removing 150 mL of pericardial effusion and obtaining tissue samples for histopathologic examination. She was discharged with analgesics.

Three weeks later, histopathological analysis confirmed an epithelioid angiosarcoma. An interdisciplinary tumor board conference recommended neoadjuvant chemotherapy and subsequent reevaluation of surgical options. On readmission, follow-up TTE showed recurrence of a moderate, circumferential pericardial effusion (17 mm in diastole) without signs of hemodynamic compromise. The patient complained of similar symptoms as before, along with general fatigue. Physical examination, vital signs, and laboratory results remained unremarkable.

Percutaneous fluoroscopy-guided needle pericardiocentesis was performed through the subxiphoid approach. Approximately 280 mL of hemorrhagic fluid was aspirated. Immediately post-procedure, no pericardial effusion was visible on TTE. Cytologic examination revealed a high cell count with neutrophils, lymphocytes, and erythrocytes. It was negative for malignant cells. We thus considered the pericardial effusion to be reactive or inflammatory. A few hours after the procedure, a central venous catheter was placed to allow the prompt initiation of chemotherapy, followed by a chest X-ray to verify its position.

**Figure 1:**
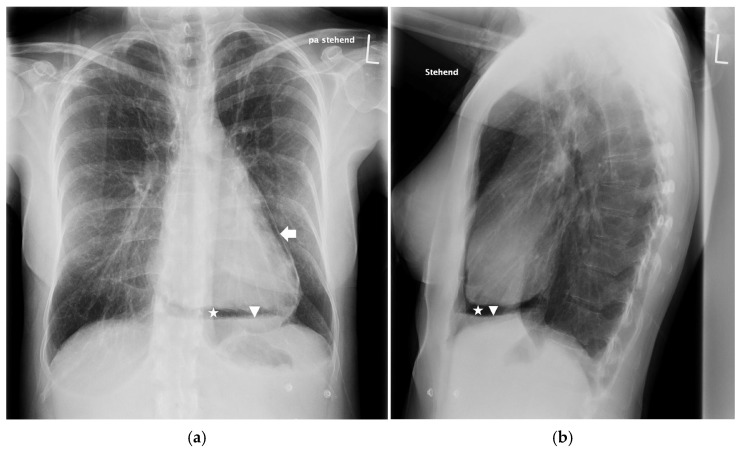
Posterior–anterior (**a**) and lateral (**b**) chest X-ray showing a radiolucent rim (star) separating the pericardium (arrow) from the cardiac silhouette with an air–fluid level (arrow head) within the pericardial space.

**Figure 2:**
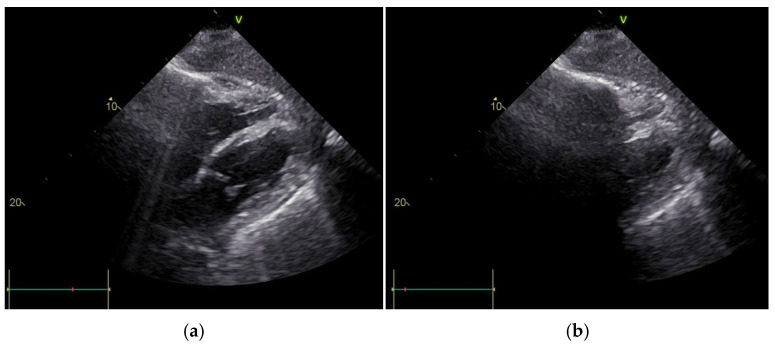
Transthoracic echocardiography subxiphoid view showing a cyclic loss of echogenicity with cardiac structures visible in diastole (**a**) and disappearing during systole (**b**).

**Figure 3:**
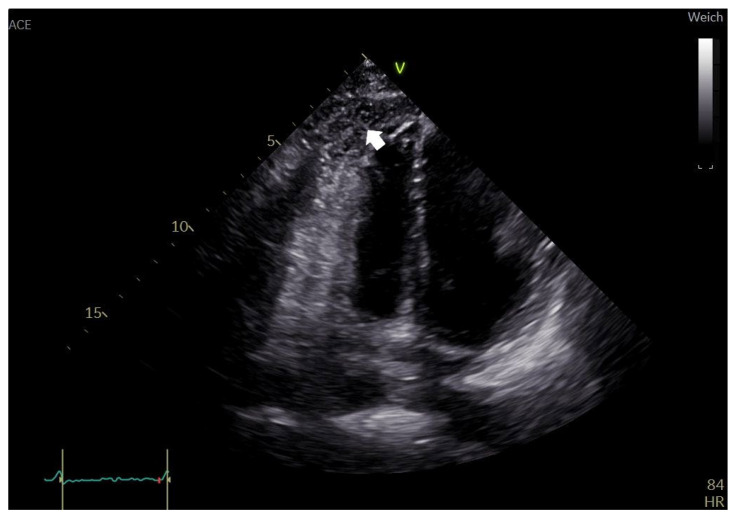
Transthoracic echocardiography apical four-chamber view showing small echogenic spots (arrow) swirling within the pericardial cavity.

**Figure 4:**
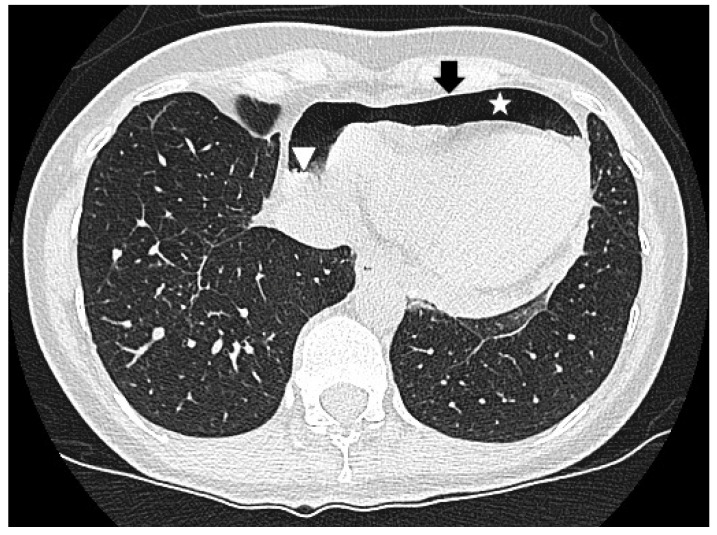
Chest computed tomography demonstrating air (star) within the pericardium (arrow) as well as an air–fluid level (arrow head), confirming pneumopericardium.

The chest X-ray revealed air entrapment between the cardiac silhouette and the pericardium, as well as an air–fluid level within the pericardial space, suggestive of pneumopericardium (Figure 1). TTE showed a cyclic loss of echogenicity occurring during systole in the subxiphoid view (Figure 2, Video S1). Furthermore, a pericardial effusion with a small amount of bright echogenic spots swirling within the pericardial cavity could be seen (Figure 3, Video S2). A CT scan of the chest confirmed pronounced pneumopericardium with consecutive RV impression, as well as a remaining pericardial effusion with an air–fluid level (Figure 4).

The patient was asymptomatic and hemodynamically stable with no signs of hemodynamic compromise on TTE. Thus, we opted for conservative management under watchful surveillance. The following days, TTE showed gradual spontaneous regression of the pneumopericardium. Chemotherapy was started three days after the pericardiocentesis, while a chest X-ray performed on the sixth day showed complete resolution of the previous findings. The patient underwent the following cycles of chemotherapy without complications.

## 3. Materials and Methods

### 3.1. Search Strategy

We conducted a systematic literature review following the Preferred Reporting Items for Systematic Reviews and Meta-Analyses (PRISMA) guidelines [11]. The PubMed database was searched from inception until January 2025 for articles reporting on iatrogenic pneumopericardium following pericardiocentesis. The keywords were combined with Boolean operators to broaden, focus, and restrict the search. Our primary search terms were “Pneumopericardium” OR “Hydropneumopericardium” AND “Pericardiocentesis”. We then performed a backward citation search by manually searching the reference lists of all included publications for additional articles that were not retrieved during the initial database search.

### 3.2. Selection Process

First, an overall screening of articles by title and abstract was carried out. Then, a full-text assessment was conducted. We selected relevant articles based on the eligibility criteria described below. One investigator contributed to the selection process, and no automation tools were used.

The inclusion criteria were as follows: Only studies involving iatrogenic pneumopericardium following pericardiocentesis were considered eligible for inclusion in this systematic review. All study types were included. Our focus of interest was iatrogenic pneumopericardium after pericardiocentesis in all its aspects: clinical manifestations, underlying causes, diagnostic tools, management options, and outcome. Studies that did not provide information on all of these aspects were also included, clearly stating when data were missing and which type of information was not available.

Exclusion criteria were as follows: We excluded studies belonging to one of the following categories: articles not available in English; reports on patients under 18 years of age; articles on pneumopericardium but unrelated to its iatrogenic and unintentional occurrence after pericardiocentesis; and reports on pneumopericardium occurring after dry pericardiocentesis performed for epicardial catheter ablation of cardiac arrhythmias.

### 3.3. Data Collection, Extraction, and Analysis

We collected the following items: first author, demographics (age and gender), causes of pericardial effusion, tamponade physiology before pericardiocentesis, method of pericardiocentesis, amount of effusion drained by pericardiocentesis, placement of a catheter for extended drainage, time between pericardiocentesis and diagnosis of pneumopericardium, imaging methods used in the assessment of pneumopericardium, modality for confirmation of the diagnosis of pneumopericardium, detection of an air–fluid level, detection of echocardiographic signs of pneumopericardium, injuries additional to pneumopericardium, clinical presentation (signs and symptoms) and hemodynamic compromise, therapy and outcome of pneumopericardium, time for pneumopericardium to resolve, and assumed reason for pneumopericardium development. Variables not reported were labeled “not reported” (NR). Data were analyzed using Microsoft Excel. Standard methods of descriptive statistics were applied. One investigator contributed to data collection and extraction.

### 3.4. Risk of Bias Assessment

All articles included in this systematic review were case reports. Two investigators independently appraised the quality of each case report by using the Joanna Briggs Institute (JBI) Critical Appraisal Checklist for Case Reports on a scale of zero to eight [12]. Case reports that received a score of four or less were considered to have low-quality evidence, and those with a score of five to six were considered to have moderate-quality evidence, while those that received a score of seven to eight were considered to have evidence of high quality. Differences in scoring were resolved through discussion and did not require a third author due to good inter-rater reliability.

## 4. Results

### 4.1. Study Selection

**Figure 5:**
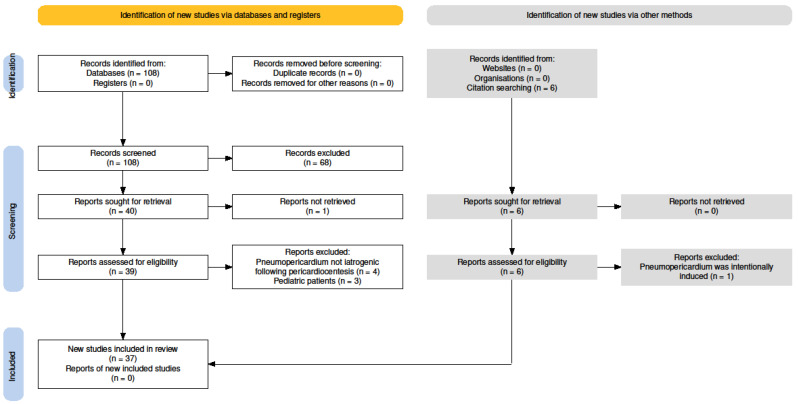
Flow diagram of the study selection process, created with the Preferred Reporting Items for Systematic Reviews and Meta-Analyses (PRISMA) Flow Diagram tool [13].

Figure 5 illustrates the flow chart of our study selection process. The search strategy initially resulted in the identification of 108 publications from the PubMed database, which all underwent screening. We excluded 68 articles after reading the titles and abstracts. Of the 40 publications sought for retrieval, 39 full-text articles were retrieved and assessed for final eligibility, while one was not successfully retrieved. Following a comprehensive review of these 39 full-text publications, an additional 7 articles were excluded for various reasons. Four cases were not related to iatrogenic pneumopericardium following pericardiocentesis, while three articles reported on pediatric patients.

Additionally, citation searching resulted in the identification of six further publications of interest. All six were retrieved and assessed for eligibility. One article was excluded because the pneumopericardium was intentionally induced. Finally, a total of 37 articles met the inclusion and exclusion criteria of this systematic review.

### 4.2. Study Characteristics

All of the articles included in the systematic review were case reports. Details of the included cases are collectively reported in Table 1, Tables S1 and S2.

Among the 37 cases examined in this review, 35.1% (13) were female. The median age was 55 years (interquartile range (IQR) of 29 years).

Of the 37 included cases, 86.5% (32) reported on the initial clinical and echocardiographic presentation prior to pericardiocentesis (Table S1); 90.6% (29 out of 32) of patients presented with diastolic RA or RV collapse, or cardiac tamponade. While 40.5% (15) of publications did not comment on the etiology of the pericardial effusion, various possible causes were reported. A tuberculous etiology was suggested in 31.8% (7 out of 22) of cases, while 27.3% (6 out of 22) suffered from metastatic disease.

Data about the pericardiocentesis procedure itself was incomplete (Table S1). While 59.5% (22 cases) did not specify the imaging modality used for procedural guidance, an echocardiography-guided approach was performed in 60% (9 out of 15) of the reported cases. An interventional radiologist performed the procedure in one report, suggesting a CT-guided pericardiocentesis [14]. The subxiphoid approach was most common (89.5% or 17 out of 19 cases where information was available), and 67.6% (25) of cases had a pericardiocentesis drainage catheter left in place for extended drainage. In 64.9% (24 cases), the authors reported on the amount of pericardial effusion drained (median 765 mL, IQR 359 mL).

Data relating to pneumopericardium was also incomplete (Table S2), and 94.6% (35) of authors did not mention auscultatory findings. The pathognomonic mill-wheel murmur termed “bruit de moulin”, caused by the churning movement of the heart within the pericardial cavity [15], was only reported in one instance.

Chest X-rays were performed in 86.5% (32) of patients for the evaluation of pneumopericardium, while CT and TTE were each conducted in 64.9% (24 patients). The diagnosis of pneumopericardium was confirmed by chest X-ray in 81% (30 patients) and CT in the remaining seven cases. Two authors stated that pneumopericardium had first been suspected on the basis of TTE findings and then confirmed by further imaging.

Of the 15 cases in which the time to diagnosis of pneumopericardium was clearly stated, the median was 72 h (IQR 166.5 h).

Only 37.8% (14) of publications specified both signs and symptoms of pneumopericardium. For cases where the information is generic (e.g., “hemodynamically stable” [16,17,18], “stable vital signs” [19,20,21], or “hemodynamic status deteriorated” [22,23]), further details are not known.

Of the 20 publications mentioning symptoms of pneumopericardium, dyspnea and chest pain were most commonly reported (45% (nine patients) and 35% (seven patients), respectively), and 20% (four patients) remained asymptomatic.

A total of 16 patients (51.6% of the 31 cases with reported data) developed hemodynamic compromise or showed signs of cardiac tamponade, while 13 (41.9%) remained stable without tamponade physiology. One publication issued contradictory statements regarding hemodynamics [24]. Another article stated that a “pressure pneumopericardium” developed and reported conservative management [25]. Of the six articles that did not report on hemodynamic stability or tamponade physiology, three cases required further interventions.

In 10.8% (four) of cases, pneumoperitoneum and pneumothorax were reported as additional complications.

Of the 37 included cases, 37.8% (14) did not report on the underlying cause for the development of pneumopericardium. Additionally, 40% (10) of the 25 publications with extended catheter drainage mentioned issues relating to the drainage system as a possible cause for pneumopericardium development. Conditions associated with increased negative intrathoracic pressure (e.g., coughing [16,26], obstructive sleep apnea [27], or increased respiratory effort [28,29]) were the second most common etiology, mentioned in 21.7% (5 out of 23) of cases.

In 64.9% (24) of cases, treatment was required for pneumopericardium. This included all cases complicated with injury additional to pneumopericardium (4 patients), as well as all patients who developed hemodynamic compromise (16 cases). Of the 24 patients who were treated, 75% (18) had initially presented with diastolic RA or RV collapse or cardiac tamponade prior to pericardiocentesis.

The median time to resolution of the pneumopericardium was seven days (IQR nine days) in 69.2% (9) of the 13 patients who were treated conservatively. Three authors reported that the pneumopericardium resolved within the following days and did not specify the exact time, while one group stated that it resolved gradually.

In 13.5% (five cases), patients were reported to have died. In one case, the patient developed pneumothorax and subcutaneous emphysema in addition to pneumopericardium after pericardiocentesis had been performed via a lateral–apical approach [14]. The patient died four hours after repeat pericardiocentesis was performed. The authors did not comment on the cause of death. In the second case, the patient remained in intensive care due to respiratory failure, despite hemodynamic improvement without evidence of pneumopericardium recurrence, and died one month after initial admission [30]. One patient died due to rapidly developing acute respiratory distress syndrome, despite pneumopericardium having been relieved after pericardial window formation [31]. One patient died 22 months after her initial visit due to metastatic lung cancer [32]. Another patient with metastatic lung cancer died six days post-procedure after being transferred to hospice [28].

We also extracted data on the role of TTE in the diagnosis of pneumopericardium following pericardiocentesis (Table 1).

In 12.5% (3) of the 24 case reports in which TTE was performed, the authors identified an air gap sign. Furthermore, one group described the TTE findings characteristic of this sign and stated that their findings were suggestive of pneumopericardium but failed to identify them as the air gap sign.

In 54.2% (13) of cases, echogenic spots or microbubbles were reported on TTE examination. However, only 38.5% (5 out of 13) identified their findings as the swirling bubbles sign, while 61.5% (8) merely described their findings without specifically referring to this sign.

Of the 13 patients with echogenic spots or microbubbles on TTE, 61.5% (8) demonstrated an air–fluid level on further imaging. The air–fluid level was described by the authors in 75% (six out of eight) of cases. In the remaining two cases, it was not mentioned, but it was visible upon our review of the provided images. An air–fluid level was observed in four additional cases (described by the authors in three cases and detected by us in one case), where TTE was performed but the swirling bubbles sign was not reported.

Poor visualization of cardiac structures due to the loss of acoustic windows was reported in 16.7% (four cases) [21,27,30,33].

**Table 1:** Echocardiography in the diagnosis of pneumopericardium following pericardiocentesis.

Article	Imaging			Air Gap Sign		Swirling Bubbles Sign		Air–Fluid Level	
	X-Ray	CT	TTE	Identified	Described	Identified	Described	Identified	Visible
Triantafyllis et al. [16]	Yes	No	Yes	No	No	No	Yes	No	No
Yilmaz et al. [14]	Yes	Yes	Yes	Yes	Yes	No	No	No	No
Mandal [34]	Yes	Yes	No	No	No	No	No	No	No
Iskander et al. [19]	No	Yes	Yes	Yes	Yes	Yes	Yes	No	On CT
Lee et al. [35]	No	Yes	No	No	No	No	No	No	No
Narins et al. [28]	Yes	No	Yes	No	No	No	No	No	No
Choi et al. [26]	Yes	No	Yes	No	No	No	Yes	On X-ray	No
Satyavolu et al. [24]	Yes	Yes	No	No	No	No	No	No	No
Zhu et al. [20]	No	Yes	Yes	No	No	Yes	Yes	No	No
Kenzaka et al. [32]	Yes	Yes	No	No	No	No	No	On CT	On X-ray
Özkartal et al. [22]	Yes	No	Yes	No	No	No	Yes	No	No
Pandey et al. [36]	Yes	Yes	Yes	No	No	No	No	On X-ray	On CT
Yuce et al. [23]	Yes	Yes	Yes	No	No	No	Yes	On X-ray	On CT
Bedotto et al. [37]	Yes	No	Yes	Yes	Yes	Yes	Yes	No	No
Delgado-Montero et al. [38]	Yes	Yes	No	No	No	No	No	On X-ray	On CT
Mullens et al. [39]	Yes	No	No	No	No	No	No	No	No
Methachittiphan et al. [40]	Yes	No	Yes	No	No	No	Yes	No	No
Alonso-Ventura et al. [41]	Yes	Yes	Yes	No	No	No	No	No	On X-ray, CT
Wakabayashi et al. [27]	No	Yes	Yes	No	No	No	No	No	No
Jansen et al. [42]	No	Yes	No	No	No	No	No	No	On CT
Bharucha et al. [17]	Yes	No	No	No	No	No	No	No	No
Kawanami et al. [43]	Yes	Yes	No	No	No	No	No	No	No
Tanabe et al. [21]	Yes	Yes	Yes	No	No	No	No	On X-ray	No
Abrahan IV et al. [44]	Yes	Yes	Yes	No	No	No	Yes	On CT	No
Adrover Lopez et al. [30]	Yes	Yes	Yes	No	No	No	No	No	No
Vijay and Joshi [45]	Yes	Yes	Yes	No	No	No	Yes	On X-ray	On CT
Ramírez Martínez et al. [18]	Yes	Yes	Yes	No	No	No	No	On X-ray	No
Shah et al. [46]	Yes	Yes	Yes	No	No	No	No	No	No
Garcia-Izquierdo et al. [47]	Yes	No	Yes	No	Yes	No	No	No	No
Lee et al. [48]	Yes	No	Yes	No	No	Yes	Yes	On X-ray	No
Peters et al. [49]	Yes	Yes	Yes	No	No	No	Yes	No	On X-ray
Planchat et al. [50]	Yes	Yes	No	No	No	No	No	On X-ray	No
Agstam et al. [29]	Yes	No	No	No	No	No	No	No	No
Mohanan Nair et al. [33]	Yes	No	Yes	No	No	No	No	No	No
Vohra et al. [51]	Yes	Yes	Yes	No	No	Yes	Yes	On X-ray	No
Varol et al. [25]	Yes	No	No	No	No	No	No	No	No
Kim et al. [31]	Yes	Yes	No	No	No	No	No	No	No

CT, computed tomography; TTE, transthoracic echocardiography.

### 4.3. Risk of Bias

Table 2 summarizes all publications’ risk of bias evaluation according to the JBI Critical Appraisal Checklist for Case Reports [12]. To summarize, 32.4% (12) of the included case reports were considered to have low-quality evidence, 48.6% (18) moderate-quality evidence, and 18.9% (7) high-quality evidence. The case reports considered to have low-quality evidence were not excluded due to meeting the necessary conditions required in our systematic review. Moreover, a limited quantity of appropriate articles constrained us to include these case reports. With a mean of 5.2, the overall quality of the cases was moderate, implying an unclear risk of bias.

The item with the fewest number of publications applying to it was item 5 (“Was the intervention(s) or treatment procedures(s) clearly described?”), with only 10 case reports receiving a score of “yes”. On the other hand, item 1 (“Were patient’s demographic characteristics clearly described?”), item 4 (“Were diagnostic tests or assessment methods and the results clearly described?”), and item 7 (“Were adverse events (harms) or unanticipated events identified and described?”) received a “yes” score in all 37 publications. The mean number of publications applying to an item was 24.1.

**Table 2:** Risk of bias evaluation according to the Joanna Briggs Institute (JBI) Critical Appraisal Checklist for Case Reports [12].

Article	Item Number and Corresponding Score								Yes	No	Unclear	N.A.	Score	Quality
	1	2	3	4	5	6	7	8						
Triantafyllis et al. [16]	Y	N	Y	Y	Y	U	Y	Y	6	1	1	0	6	Fair
Yilmaz et al. [14]	Y	U	Y	Y	U	U	Y	Y	5	0	3	0	5	Fair
Mandal [34]	Y	Y	U	Y	U	Y	Y	Y	6	0	2	0	6	Fair
Iskander et al. [19]	Y	U	Y	Y	Y	Y	Y	Y	7	0	1	0	7	Good
Lee et al. [35]	Y	U	Y	Y	U	U	Y	Y	5	0	3	0	5	Fair
Narins et al. [28]	Y	U	U	Y	U	U	Y	Y	4	0	4	0	4	Poor
Choi et al. [26]	Y	Y	Y	Y	U	Y	Y	Y	7	0	1	0	7	Good
Satyavolu et al. [24]	Y	Y	Y	Y	Y	N	Y	Y	7	1	0	0	7	Good
Zhu et al. [20]	Y	U	Y	Y	U	Y	Y	Y	6	0	2	0	6	Fair
Kenzaka et al. [32]	Y	Y	Y	Y	N	U	Y	Y	6	1	1	0	6	Fair
Özkartal et al. [22]	Y	U	Y	Y	N	U	Y	U	4	1	3	0	4	Poor
Pandey et al. [36]	Y	N	N	Y	N	Y	Y	Y	5	3	0	0	5	Fair
Yuce et al. [23]	Y	N	N	Y	U	U	Y	Y	4	2	2	0	4	Poor
Bedotto et al. [37]	Y	Y	U	Y	N	U	Y	Y	5	1	2	0	5	Fair
Delgado-Montero et al. [38]	Y	N	U	Y	U	N	Y	U	3	2	3	0	3	Poor
Mullens et al. [39]	Y	N	Y	Y	N	N	Y	N	4	4	0	0	4	Poor
Methachittiphan et al. [40]	Y	N	N	Y	N	Y	Y	N	4	4	0	0	4	Poor
Alonso-Ventura et al. [41]	Y	Y	Y	Y	U	U	Y	Y	6	0	2	0	6	Fair
Wakabayashi et al. [27]	Y	N	N	Y	N	Y	Y	Y	5	3	0	0	5	Fair
Jansen et al. [42]	Y	N	N	Y	U	U	Y	U	3	2	3	0	3	Poor
Bharucha et al. [17]	Y	U	Y	Y	U	U	Y	Y	5	0	3	0	5	Fair
Kawanami et al. [43]	Y	Y	U	Y	Y	U	Y	U	5	0	3	0	5	Fair
Tanabe et al. [21]	Y	N	U	Y	U	U	Y	N	3	2	3	0	3	Poor
Abrahan IV et al. [44]	Y	Y	Y	Y	Y	U	Y	Y	7	0	1	0	7	Good
Adrover Lopez et al. [30]	Y	Y	Y	Y	N	U	Y	Y	6	1	1	0	6	Fair
Vijay and Joshi [45]	Y	U	Y	Y	N	Y	Y	Y	6	1	1	0	6	Fair
Ramírez Martínez et al. [18]	Y	N	U	Y	U	Y	Y	Y	5	1	2	0	5	Fair
Shah et al. [46]	Y	Y	Y	Y	Y	Y	Y	Y	8	0	0	0	8	Good
Garcia-Izquierdo et al. [47]	Y	N	U	Y	N	U	Y	N	3	3	2	0	3	Poor
Lee et al. [48]	Y	Y	Y	Y	Y	Y	Y	Y	8	0	0	0	8	Good
Peters et al. [49]	Y	U	U	Y	Y	U	Y	Y	5	0	3	0	5	Fair
Planchat et al. [50]	Y	U	N	Y	U	N	Y	Y	4	2	2	0	4	Poor
Agstam et al. [29]	Y	U	Y	Y	U	N	Y	U	4	1	3	0	4	Poor
Mohanan Nair et al. [33]	Y	U	Y	Y	Y	Y	Y	U	6	0	2	0	6	Fair
Vohra et al. [51]	Y	N	Y	Y	Y	Y	Y	Y	7	1	0	0	7	Good
Varol et al. [25]	Y	N	Y	Y	N	N	Y	N	4	4	0	0	4	Poor
Kim et al. [31]	Y	U	U	Y	N	Y	Y	Y	5	1	2	0	5	Fair
**Number of articles applying to this item**	37	11	21	37	10	14	37	26						

Y, yes; N, no; U, unclear; N.A., not applicable. Items/questions from the JBI Critical Appraisal Checklist for Case Reports: 1. Were patient’s demographic characteristics clearly described? 2. Was the patient’s history clearly described and presented as a timeline? 3. Was the current clinical condition of the patient on presentation clearly described? 4. Were diagnostic tests or assessment methods and the results clearly described? 5. Was the intervention(s) or treatment procedure(s) clearly described? 6. Was the post-intervention clinical condition clearly described? 7. Were adverse events (harms) or unanticipated events identified and described? 8. Does the case report provide takeaway lessons?

## 5. Discussion

This comprehensive systematic review evaluated the clinical presentation, underlying causes, diagnosis, and management of patients with iatrogenic pneumopericardium following pericardiocentesis.

Pericardiocentesis carries a 4–10% risk of complications depending on the setting (i.e., elective vs. emergency), physician experience, and type of procedural monitoring [8]. While fluoroscopic guidance further improves the safety of echocardiography-guided pericardiocentesis [52], both echocardiography-guided [53] and fluoroscopy-guided [54] procedures have shown high success rates and low complication rates. Likewise, leaving an intrapericardial catheter for extended drainage is safe and has been shown to decrease effusion recurrences in comparison to simple pericardiocentesis [55,56].

Pneumopericardium has been listed as an uncommon complication of pericardiocentesis [8,9]. To date, there is a lack of data on its frequency. No cases of pneumopericardium were reported in publications covering 977 patients with 1127 echocardiography-guided pericardiocentesis procedures [53], 333 patients undergoing 386 fluoroscopy-guided procedures [54], and 571 patients with 635 CT-guided procedures [57]. A significant proportion of patients with pericardial effusion remain asymptomatic and are unexpectedly diagnosed when imaging is performed for other indications [10]. Given the similarity in clinical presentation and the rarity of pneumopericardium compared to pericardial effusion, it is reasonable to speculate that a considerable number of cases may remain undetected. This assumption is supported by various publications, which report that patients with pneumopericardium frequently remain asymptomatic [1,19,58]. However, in our systematic review of the literature, more than half of patients with available information (51.6% or 16 out of 31 cases) developed hemodynamic compromise or showed signs of cardiac tamponade (Table S2). Additionally, three cases in which the authors did not report on hemodynamic stability required decompression, indicating hemodynamic impact. We attribute this discrepancy to publication bias, where cases with complications are more likely to be diagnosed and reported.

The authors of the included cases mentioned various underlying causes for the development of pneumopericardium (Table S2). Issues relating to the drainage system in procedures with extended drainage were the most frequently mentioned reason (40% of cases with extended drainage). In six further cases, the diagnosis of pneumopericardium was made after the removal of the drainage system, indicating it may have played a role in causing the complication. Though information on initial presentation prior to pericardiocentesis was only available for a subset of 32 cases, a majority of patients (90.6% or 29 out of 32) presented with diastolic RA or RV collapse or cardiac tamponade (Table S1). This may have been a contributing factor, as the performance of pericardiocentesis in emergency situations carries a higher likelihood of complications [8].

In our patient, CT ruled out a fistulous communication. Central venous catheterization cannot be entirely excluded as a possible cause of the pneumopericardium. It has been reported once in the literature after several unsuccessful attempts at the placement of a central venous catheter were made [59]. While TTE immediately after pericardiocentesis showed no signs of pericardial effusion or pneumopericardium, central venous catheterization was uncomplicated and performed by an experienced intensive care physician. We hypothesize that air was induced due to forced coughing or deep inspiration during the procedure, as has been previously reported [16,26,29].

Correct puncture direction and depth, closed drainage system connections at the time of puncture, and the creation of an airtight seal by application of surgical gel to the skin surrounding the drainage are essential for a successful procedure. Additionally, avoiding negative intrapericardial pressure (e.g., strenuous aspiration, deep breaths, or coughing) during open needle access in pericardiocentesis or procedures involving extended catheter drainage is paramount [5].

Our systematic review also analyzed the diagnostic procedures involved in the work-up of pneumopericardium, focusing on the role of TTE in the diagnosis of pneumopericardium following pericardiocentesis (Table 1).

The “bruit de moulin” murmur was only reported in one case. The relative proportions of fluid and air, the specific gravity of the fluid, and pericardial adhesions have been proposed as factors in the occurrence of this pathognomonic finding [60], and this may explain why it was rarely encountered.

While chest X-ray and CT are the imaging modalities of choice for the diagnosis of pneumopericardium and were performed in all cases, TTE was part of the diagnostic work-up in approximately two-thirds (64.9%) of cases. Two TTE findings are often cited in the literature as being characteristic of pneumopericardium. The “air gap sign” is described as the cyclic disappearance of cardiac structures during systole due to the appearance of air as it circulates within the pericardial cavity [37,61,62]. With the reduction in chamber volume during systolic contraction, air within the pericardium accumulates anteriorly to interface between the heart and the transducer. This results in the loss of a part of the cardiac image coinciding with each systole in two-dimensional and M-mode echocardiography. Chamber expansion during diastole results in air displacement. The cardiac structures previously obscured by air thereby reappear. Examination during held expiration may help discriminate interference caused by overlying lung tissue. As the air gap sign has been described in both pneumopericardium and pneumomediastinum [61], a means of differentiating was described by the inability to visualize cardiac structures from the subxiphoid view in pneumopericardium. In pneumomediastinum, anatomy is well visualized from this view due to the fact that the heart remains in contact with the diaphragm without air impairing visualization [63,64].

The second frequently mentioned finding is the “swirling bubbles sign”, described as the continuous churning movement of small echogenic spots within the pericardial cavity in hydropneumopericardium, when both fluid and air are present in the pericardium [58,65]. The swirling bubbles sign is thought to be caused by air bubbles that are formed by continuous agitation of the air–fluid interface and are seen swirling within the pericardial fluid. However, the subset of patients (38.5% or 5 out of 13) who exhibited echogenic spots or microbubbles on TTE but lacked an air–fluid level on further imaging, as well as the four additional cases where an air–fluid level was observed and TTE was performed but the swirling bubbles sign was not reported, highlight the need for further research on its pathophysiology.

Only a minority of authors (16.7% or 4 out of 24 cases in which TTE was performed) reported the air gap sign. While echogenic spots or microbubbles were observed on TTE in approximately half of all cases (54.2% or 13 cases), only a subset (38.5% or 5 out of 13) identified their findings as the swirling bubbles sign. In total, among the 24 cases in which TTE was conducted, roughly three-quarters failed to describe the air gap and swirling bubbles signs as characteristic features of pneumopericardium (75% or 18 cases and 70.8% or 17 cases, respectively). The mean quality of evidence for these publications (i.e., 5.3 and 5.2, respectively) was in line with the overall mean quality of included cases. Of the four cases with echocardiographic findings of an air gap sign, two did not comment on or mention the swirling bubbles sign. Similarly, approximately two-thirds (69.2% or 9 out of 13) of the cases with echocardiographic findings of a swirling bubbles sign did not comment on or mention the air gap sign. This highlights a general unawareness regarding these non-validated “characteristic” echocardiographic findings. Furthermore, their recognition and identification are also challenging. This is demonstrated by four cases in which TTE had been performed, where the air gap and/or swirling bubbles signs were noted as characteristic of pneumopericardium but were not reported in the findings [20,28,30,48].

While both the air gap (Figure 2) and swirling bubbles signs (Figure 3), as well as an air–fluid level, were visible in our patient, the X-ray (Figure 1) had alerted us to the presence of air within the pericardium before performing TTE. The identification of these echocardiographic signs, as such, was made in hindsight, after the diagnosis of pneumopericardium had been established. Solely depending on TTE would have made recognition difficult.

Following pericardiocentesis, serial echocardiographic follow-ups have been recommended to evaluate pericardial effusion recurrence [52]. While TTE is considered the first-line imaging modality for evaluating pericardial diseases [52,66], with chest X-ray having a limited role [67], our results suggest that echocardiography may not be suitable for detecting pneumopericardium. The sensitivity and specificity of the air gap and swirling bubbles signs for diagnosing pneumopericardium have yet to be studied. Furthermore, identifying these signs can be especially challenging in asymptomatic patients, where pneumopericardium may not be suspected. Therefore, a chest X-ray should be considered in cases of suspected pneumopericardium. However, TTE may be useful for monitoring patients once the diagnosis has been confirmed and for guiding the timing of a possible intervention [68].

It is widely stated that iatrogenic pneumopericardium following pericardiocentesis generally resolves spontaneously and is self-limiting [19,26,39,48]. In contrast, our systematic review found that approximately two-thirds (64.9% or 24 out of 37) of patients required an intervention (Table S2). As mentioned above, we attribute this finding to publication bias.

Pneumopericardium carries a poor prognosis. In a review of 252 patients, the condition was associated with a 57% mortality [1]. However, the varying underlying causes of pneumopericardium (e.g., 62% due to trauma and 25% due to infection in contiguous organs with fistulous tracts to the pericardial sac), as well as heterogeneous patient population (e.g., tension pneumopericardium associated with assisted ventilation in neonates in 76 of 94 cases), preclude a direct comparison to the outcomes of the patients included in our review. Here, the mortality was 13.5% (five patients), with four of these deaths being unrelated to pneumopericardium. Thus, the mortality of iatrogenic pneumopericardium following pericardiocentesis appears to be lower than that associated with other causes of pneumopericardium.

The main limitation of this study is that, regarding iatrogenic pneumopericardium following pericardiocentesis, only case reports have been published—not even case series. The 37 publications included in our review were considered to have an overall evidence of moderate quality according to the JBI Critical Appraisal Checklist for Case Reports [12], with a mean score of 5.2 out of 8 (Table 2). However, the hypothetical exclusion of items 1 (all cases reported patient age and sex and were scored “yes”), 4 (all cases performed chest X-ray and/or CT to confirm the diagnosis of pneumopericardium and were scored “yes”), 7 (all cases identified the adverse event, namely pneumopericardium, and were scored “yes”), and 8 (most cases summarized the key lessons and were scored “yes”) results in a mean score of 1.5 out of 4. The reason for this poor appraisal is the inconsistent reporting of several key components in the cases reviewed. This includes crucial information such as the method of pericardiocentesis and the clinical presentation of patients post-procedure, which limits direct comparisons between cases. Furthermore, the limited number of case reports prevents drawing general conclusions about pneumopericardium.

## 6. Conclusions

We present a rare case of pericardiocentesis complicated by iatrogenic pneumopericardium in a patient with primary cardiac angiosarcoma. Conservative management of the asymptomatic patient under careful surveillance led to the spontaneous resolution of pneumopericardium after six days. Our case highlights the importance of considering pneumopericardium as a possible complication of pericardiocentesis.

The overall evidence of the quality of the 37 case reports included in our systematic review was considered moderate according to the JBI Critical Appraisal Checklist for Case Reports, as crucial information was inconsistently reported.

Approximately half of the patients with pneumopericardium developed hemodynamic compromise or exhibited signs of tamponade physiology, while roughly two-thirds required decompressive therapy. However, we speculate that there is a high proportion of asymptomatic and, therefore, undiagnosed cases.

Our results highlight the challenges of diagnosing pneumopericardium using TTE. Due to the rarity of this complication, the recognition and accurate interpretation of its TTE findings is difficult. While its scarcity may not justify the routine use of imaging with ionizing radiation in all patients undergoing pericardiocentesis, urgent multi-plane chest X-ray or CT should be employed for further evaluation if pneumopericardium is suspected. Prospective trials that perform regular imaging on all patients undergoing pericardiocentesis could provide valuable insights into incidence rates.

## Data Availability

No new data were created or analyzed in this study. Data sharing is not applicable to this article.

## Supplementary Materials

The following supporting information can be downloaded at: https://www.mdpi.com/article/10.3390/jcdd12070246/s1, Table S1: Data on patient characteristics and pericardiocentesis procedure; Table S2: Data on pneumopericardium; Video S1: Air gap sign; Video S2: Swirling bubbles sign.

## Abbreviations

The following abbreviations are used in this manuscript:

CT	Computed tomography
JBI	Joanna Briggs Institute
PRISMA	Preferred Reporting Items for Systematic Reviews and Meta-Analyses
RV	Right ventricle
TTE	Transthoracic echocardiography
