# Supplementary material for: Iatrogenic Pneumopericardium After Pericardiocentesis: A Systematic Review and Case Report
Source: J Cardiovasc Dev Dis. 2025 Jun 26;12(7):246. doi: 10.3390/jcdd12070246 (PMC12295399; doi:10.3390/jcdd12070246)
Supplement: Supplementary file 1 [file jcdd-12-00246-s001.zip › jcdd-3611412-supplementary.pdf]

Journal of
Cardiovascular
Development and Disease

MDPI

Systematic Review

Iatrogenic Pneumopericardium After Pericardiocentesis: A Systematic Review and Case Report

Andreas Merz[1,2,3,*], Hong Ran[4], Cheng-Ying Chiu[1,2,3], Henryk Dreger[1,2,3], Daniel Armando Morris[1,2,3] and Matthias Schneider-Reigbert[1,2,3]

1 Deutsches Herzzentrum der Charité, Department of Cardiology, Angiology and Intensive Care Medicine, Berlin, Germany.
2 DZHK (German Center for Cardiovascular Research), partner site Berlin, Germany.
3 Charité – Universitätsmedizin Berlin, corporate member of Freie Universität Berlin and Humboldt-Universität zu Berlin, Berlin, Germany.
4 Department of Echocardiography, Nanjing First Hospital, Nanjing Medical University, Nanjing, China.
* Correspondence: andreas.merz@dhzc-charite.de

Table S1. Data on patient characteristics and pericardiocentesis procedure

Article	Patient		Pericardiocentesis				
	Gender	Age	Clinical context		Procedure	Drainage	
			Reason of pericardial effusion	Signs of tamponade		Extended drainage	Amount (ml)
Triantafyllis et al. [16]	M	48	Idiopathic	Yes	Fluoroscopy-guided subxiphoid	No	1000
Yilmaz et al. [14]	M	56	Coronavirus	Yes	NR	Yes	750
Mandal [34]	F	48	Unclear, history of tuberculosis	No	NR	No	800
Iskander et al. [19]	M	46	Uremia	Yes	Echocardiography-guided subxiphoid	Yes	1600
Lee et al. [35]	M	70	NR	Yes	Subxiphoid	Yes	700
Narins et al. [28]	F	73	NR. History of metastatic disease	Yes	Echocardiography-guided	Yes	550
Choi et al. [26]	M	20	Tuberculosis	Yes	Subxiphoid	Yes	>1000
Satyavolu et al. [24]	M	86	Pericarditis	Yes	Echocardiography-guided subxiphoid	Yes	1200
Zhu et al. [20]	M	69	Hypothyroidism	Yes	Subxiphoid	Yes	200
Kenzaka et al. [32]	F	74	Metastatic disease	Yes	NR	No	NR
Özkartal et al. [22]	F	73	NR. History of metastatic disease	No	NR	Yes	NR
Pandey et al. [36]	M	21	Tuberculosis	NR	NR	No	NR
Yuce et al. [23]	M	54	NR	NR	Subxiphoid	No	800
Bedotto et al. [37]	M	29	Metastatic disease	Yes	NR	Yes	NR
Delgado-Montero et al. [38]	M	80	NR	Yes	Echocardiography-guided	No	NR
Mullens et al. [39]	F	30	NR	Yes	NR	No	600
Methachittiphan et al. [40]	F	69	Idiopathic	NR	NR	No	NR
Alonso-Ventura et al. [41]	M	90	NR	Yes	Subxiphoid	Yes	1350
Wakabayashi et al. [27]	M	62	Catheter ablation	Yes	NR	Yes	NR
Jansen et al. [42]	M	79	Tacrolimus	Yes	Subxiphoid	No	NR
Bharucha et al. [17]	M	21	Coronavirus	Yes	Fluoroscopy-guided	Yes	750
Kawanami et al. [43]	F	93	NR. History of scleroderma	Yes	Echocardiography-guided apical	Yes	NR
Tanabe et al. [21]	F	47	Graft-vs.-host disease	Yes	Subxiphoid	Yes	400

Abrahan IV et al. [44]	M	28	Tuberculosis with superinfection	Yes	Echocardiography-guided subxiphoid	Yes	625
Adrover Lopez et al. [30]	M	70	NR. History of metastatic disease	Yes	NR	Yes	1300
Vijay and Joshi [45]	F	20	Tuberculosis	NR	NR	No	NR
Ramírez Martínez et al. [18]	M	80	NR	No	Subxiphoid	Yes	NR
Shah et al. [46]	M	55	NR	Yes	Echocardiography-guided subxiphoid	Yes	780
Garcia-Izquierdo et al. [47]	M	55	NR. History of metastatic disease	Yes	NR	Yes	600
Lee et al. [48]	M	91	Tuberculosis	Yes	Fluoroscopy-guided subxiphoid	Yes	640
Peters et al. [49]	F	47	Scleroderma	Yes	Echocardiography-guided subxiphoid	No	800
Planchat et al. [50]	M	52	Chronic pericardial effusion	Yes	Subxiphoid	Yes	860
Agstam et al. [29]	F	25	NR	Yes	Fluoroscopy-guided	No	1000-1500
Mohanan Nair et al. [33]	M	65	Catheter ablation	Yes	Fluoroscopy-guided subxiphoid	Yes	540
Vohra et al. [51]	M	35	Tuberculosis	Yes	Echocardiography-guided subxiphoid	Yes	1500
Varol et al. [25]	F	85	NR	NR	NR	Yes	NR
Kim et al. [31]	F	44	NR	Yes	NR	Yes	NR

M, Male; F, Female; NR, Not Reported.

Table S2. Data on pneumopericardium, including diagnosis, underlying cause, clinical presentation, treatment, as well as outcome

Article	Pneumopericardium									
	Diagnosis		Cause	Clinical presentation				Therapy	Outcome	Time to resolution in conservative therapy
	Time to	Method		Signs of tamponade	Haemo-dynamic compromise	Symptoms	Other			
Triantafyllis et al. [16]	24 hrs.	X-ray	Drainage system and negative intrathoracic pressure	NR	No	NR	NR	Conservative	Resolved	Gradually
Yilmaz et al. [14]	3 hrs.	X-ray	Apical-lateral approach	NR	NR	Chest pain	Pneumothorax, subcutaneous emphysema	Pericardiocentesis	Death 4 hrs. later	N/A
Mandal [34]	Next day	X-ray	NR	NR	Yes	Pleuritic chest pain	NR	Pericardiocentesis	Resolved	N/A
Iskander et al. [19]	10d	CT (suspected on TTE)	Drainage system	No	No	Pleuritic chest pain	NR	Conservative	Resolved	Following days
Lee et al. [35]	NR. Suggestive of hrs.	CT	Drainage system	Yes	Yes	NR	NR	Air evacuated through extended drainage catheter	Transferred to long term care hospital	N/A
Narins et al. [28]	Next day	X-ray	Negative intrathoracic pressure	No	NR	Dyspnea	NR	Air evacuated through extended drainage catheter	Death 6d later	N/A
Choi et al. [26]	5d	X-ray	Negative intrathoracic pressure	No	No	Pleuritic chest pain	NR	Conservative	Resolved	5d
Satyavolu et al. [24]	NR. Drain removed after 2d, then hissing	X-ray	Bowel distention and elevated left hemidiaphragm	NR	Unclear	Unclear	Transverse colon perforation,	Pericardiocentesis, pericardial window	Resolved	N/A

	sound was heard and diagnosis confirmed.						pneumoperiton eum			
Zhu et al. [20]	2d	CT	Pleural-pericardial fistula	No	No	Asympto-matic	NR	Conservative	Resolved	1 mo.
Kenzaka et al. [32]	NR. Suggestive of hrs.	X-ray	Puncture without continuous suction	NR	Yes	NR	NR	Pericardiocentesis	Death 22 mo. later	N/A
Özkartal et al. [22]	Next day	X-ray	Drainage system	NR	Yes	Clinical condition worsened progressively	NR	Pericardiocentesis	NR	N/A
Pandey et al. [36]	1 mo.	X-ray	Drainage system	Yes	Yes	Dyspnea, chest pain	Pulsus paradoxus, elevated jugular venous pressure	Pericardiocentesis, pericardectomy	Resolved	N/A
Yuce et al. [23]	10d	X-ray	NR	NR	Yes	NR	NR	Pericardiocentesis	NR	N/A
Bedotto et al. [37]	NR. Drain removed after several days, then diagnosis was confirmed.	X-ray	NR	NR	Yes	NR	Bruit de moulin murmur	Thoracotomy and pericardial stripping	NR	N/A
Delgado-Montero et al. [38]	3d	X-ray	NR	NR	NR	NR	NR	Conservative	Resolved	3d
Mullens et al. [39]	3 hrs.	X-ray	NR	NR	NR	NR	NR	Conservative	Resolved	Following days
Methachittiphan et al. [40]	1d	X-ray	NR	Yes	Yes	Dyspnea	Pulsus paradoxus, elevated	Surgical pericardial drainage	NR	N/A

							jugular venous pressure			
Alonso-Ventura et al. [41]	NR. Drain removed, then diagnosis was confirmed.	X-ray	NR	No	No	NR	Pulsus paradoxus	Conservative	Resolved	4 wk.
Wakabayashi et al. [27]	hrs.	CT	Negative intrathoracic pressure	Yes	Yes	Chest pain	NR	Air evacuated through extended drainage catheter	NR	N/A
Jansen et al. [42]	12d	CT	NR	Yes	Yes	NR	Pneumo-peritoneum	Minithoracotomy, laparotomy	Resolved	N/A
Bharucha et al. [17]	3 hrs.	X-ray	Drainage system	NR	No	NR	NR	Air evacuated through extended drainage catheter	NR	N/A
Kawanami et al. [43]	NR	CT	Pleura-pericardial fistula	NR	No	NR	Pneumothorax	Air evacuated through extended drainage catheter	Resolved	N/A
Tanabe et al. [21]	Drain removed after 2d, following day diagnosis was confirmed.	X-ray	Needle tract created fistula with check-valve mechanism	NR	No	NR	NR	Conservative	Resolved	7d
Abrahan IV et al. [44]	5d	X-ray	NR	NR	No	Dyspnea	NR	Pericardiectomy	Resolved	N/A
Adrover Lopez et al. [30]	NR. Suggestive of hrs.	X-ray	Drainage system	Yes	Yes	NR	NR	Air evacuated through extended drainage catheter	Death 1 mo. later	N/A
Vijay and Joshi [45]	NR	X-ray	NR	Yes	Yes	Dyspnea	Diminished heart sounds,	Surgical drainage	Resolved	N/A

							pulsus paradoxus			
Ramírez Martínez et al. [18]	NR. Drain removed after 24 hrs., then diagnosis was confirmed.	X-ray	NR	NR	No	Asympto-matic	NR	Conservative	Resolved	Following days
Shah et al. [46]	NR. Drain removed next day, then diagnosis was confirmed.	CT	NR	No	No	Asympto-matic	NR	Conservative	Resolved	2 wk.
Garcia-Izquierdo et al. [47]	NR. Drain removed after 3d then diagnosis was confirmed.	X-ray (suspected on TTE)	NR	NR	NR	Asympto-matic	NR	Conservative	Resolved	1 wk.
Lee et al. [48]	Drain was found removed from patient during sleeping on next day, then 2d later diagnosis was confirmed.	X-ray	Drainage system	Yes	Yes	Dyspnea, orthopnea	NR	Pericardiocentesis	NR	N/A
Peters et al. [49]	Next day	X-ray	Needle tract created remained open due to nature of skin and subcutaneous tissue (scleroderma)	No	No	NR	NR	Conservative	Resolved	4d
Planchat et al. [50]	NR	X-ray	Unusual path of drain	NR	NR	NR	NR	Air evacuated	NR	N/A

								through extended drainage catheter		
Agstam et al. [29]	During procedure	X-ray	Negative intrathoracic pressure	NR	NR	NR	NR	Pericardiocentesis was continued	NR	N/A
Mohanan Nair et al. [33]	5 min	X-ray	Side port of sheath accidentally opened	NR	Yes	Dyspnea	NR	Air evacuated through extended drainage catheter	Resolved	N/A
Vohra et al. [51]	3d	X-ray	Drainage system	NR	Yes	Dyspnea, chest pain	NR	Air evacuated through extended drainage catheter	Resolved	N/A
Varol et al. [25]	NR	X-ray	Drainage system	Pressure pneumo-pericardiu m	NR	NR	NR	Conservative	NR	NR
Kim et al. [31]	NR. Drain removed after 12d, then diagnosis was confirmed.	X-ray	NR	Yes	Yes	Dyspnea	NR	Pericardial window	Death	N/A

CT, Computed Tomography; NR, Not Reported; N/A, Not Applicable.
